# Natural Steroidal Lactone Induces G1/S Phase Cell Cycle Arrest and Intrinsic Apoptotic Pathway by Up-Regulating Tumor Suppressive miRNA in Triple-Negative Breast Cancer Cells

**DOI:** 10.3390/metabo13010029

**Published:** 2022-12-24

**Authors:** Mohd Shuaib, Kumari Sunita Prajapati, Sanjay Gupta, Shashank Kumar

**Affiliations:** 1 Molecular Signaling & Drug Discovery Laboratory, Department of Biochemistry, Central University of Punjab, Guddha, Bathinda 151401, Punjab, India; 2 Department of Urology, Nutrition, Pharmacology and Pathology, Case Western Reserve University, Cleveland, OH 44106, USA

**Keywords:** Withania A, anticancer, apoptosis, phytochemical, MiRNA, sequencing

## Abstract

Triple-negative breast cancer (TNBC) is an aggressive subtype of breast cancer with minimal treatment options. In the present work, Withaferin A (WA), a natural steroidal lactone found in *Withania somnifera* (Solanaceae), was studied to deduce the miRNA expression modulation mediated anticancer mode of action in TNBC cells. Small RNA next generation sequencing (NGS) of WA (2 µM) and vehicle (0.1% DMSO)-treated MDA-MB-231 cells revealed a total of 413 differentially expressed miRNAs (DEMs) and demonstrated that WA potentially up-regulates the miR-181c-5p, miR-15a-5p, miR-500b-5p, miR-191-3p, and miR-34a-5p and down-regulates miR-1275, miR-326, miR-1908-5p, and miR-3940-3p among total DEMs. The NGS and qRT-PCR expression analysis revealed a significantly higher expression of miR-181c-5p among the top 10 DEMs. Predicted target genes of the DEMs showed enrichment in cancer-associated gene ontology terms and KEGG signaling pathways. Transient up-expression of mir-181c-5p showed a time-dependent decrease in MDA-MB-231 and MDA-MB-453 cell viability. Co-treatment of miR-181c-5p mimic and WA (at varying concentration) down-regulated cell cycle progression markers (CDK4 and Cyclin D1) at mRNA and protein levels. The treatment induced apoptosis in MDA-MB-231 cells by modulating the expression/activity of Bax, Bcl2, Caspase 3, Caspase 8, Caspase 3/7, and PARP at mRNA and protein levels. Confocal microscopy and Annexin PI assays revealed apoptotic induction in miRNA- and steroidal-lactone-treated MDA-MB-231 cells. Results indicate that the Withaferin A and miRNA mimic co-treatment strategy may be utilized as a newer therapeutic strategy to treat triple-negative breast cancer.

## 1. Introduction

Triple-negative breast cancer (TNBC) is a clinical aggressive subtype of breast cancer (BCa) that is defined by lack of estrogen (ER), progesterone (PR), and human epidermal growth factor receptor 2 (HER2) [1]. It affects younger women and is diagnosed at later stage(s) (advanced stage) with a higher proliferative index, histological grade, and visceral metastasis. Its prevalence rate is 15–20% among all the subtypes of BCa worldwide [1,2]. TNBC patients show higher rates of reoccurrence and develop frequent visceral metastasis compared to other subtypes of BCa. TNBC developed drug resistance against standard chemotherapy, and hormone therapy, which is related to a lower five-year survival rate in comparison to other BCa subtypes. Currently, there are two main chemotherapy agents (Taxan and anthracyclin) that are the only option to treat TNBC, but TNBC patients frequently develop resistance against these cytotoxic agents by evading apoptosis and other mechanisms [1,2]. Therefore, targeting the evasion of apoptosis has its therapeutic potential in TNBC. Apoptosis is the key process of programmed cell death that is crucial for maintaining the cell populations in tissues. However, it is largely dysregulated in human malignancies that fall under the main hallmark of cancer that are resistant to cell death [2]. The inappropriate and dysregulated apoptosis event in TNBC patients is well reported and is responsible for the poor outcome of the chemotherapy [3,4]. MicroRNAs (miRNAs) are small non-coding RNAs that generally exert their biological function by targeting more than one target gene, which indicates their regulatory potential [5,6]. MicroRNAs regulate expression of the gene(s) involved in tumor hallmarks, such as apoptosis evasion. In the past two decades, accumulated evidence has indicated that miRNAs play oncogenic and tumor-suppressive roles in TNBC by inhibiting or inducing apoptosis [7].

Natural products possess potential pharmaceutical agents and are currently drugs of choice due to their lower toxicity, decreased side-effects, and cost-effectiveness [8,9,10,11,12,13,14]. *Withania somnifera* (Solanaceae) is an Indian traditional medicinal plant known for its anticancer potential. *Withania* spp. are well known for the presence of a potential group of natural compounds known as withanolide. Withanolide is a 28 carbon phytocompound possessing oxidized carbon at the C22 and C26 positions, which results in the formation of a lactone ring [15]. Withaferin A (WA) was the first identified withanolide isolated from *W. somnifera* and is the main pharmacologically active phytochemical of the plant. WA possesses anticancer activity (in vitro and in vivo) against different cancers by altering cellular proliferation, differentiation, metastasis, angiogenesis, invasion, and drug-resistance at the biochemical and molecular levels [16]. Different reports showed that WA targets TNBC cells by targeting cell death, cell cycle, proliferation, metastasis, and apoptosis [17,18] Recently, Kim et al. (2020) reported the metabolic alteration potential of WA in in vitro and in vivo cancer experimental models [19]. Although WA has been suggested as an attractive phyto-pharmaceutical agent against TNBC cells, its anticancer effect by modulating miRNA expression in cancer cells has not yet been reported. Due to the absence of a validated molecular therapeutic target, identification of WA modulated miRNA(s) could be used to target TNBC alone or in combination with therapeutic agents. Keeping these facts in mind, the present study was designed to study the underlying mechanism of WA-mediated apoptosis by altering miRNA expression in the TNBC in vitro model.

## 2. Material and Methods

### 2.1. Cell Lines 

Human breast cancer cell lines MDA-MB-231 and MDA-MB-453 were purchased from the National Centre for Cell Sciences, Pune, India. MCF-10A cells were procured from ATCC, USA. The cells were cultured in Dulbecco’s Modified Eagle Medium (DMEM) media and supplemented with 10% heat-inactivated fetal bovine serum (FBS), 0.1 mg/mL of streptomycin, and 100 units/mL of penicillin at 37 °C in a humidified atmosphere with 5% CO_2_ [12].

### 2.2. Cytotoxicity Assay 

Withaferin A (WA) was procured from Sigma-Aldrich (89910-10MG) and dissolved in DMSO (0.1%). The cell proliferation inhibition potential of WA was studied using an MTT assay as per the method described elsewhere [20]. Briefly, the cells (1 × 10^4^ cells/well) were seeded in the 96-well microtiter plates in triplicate. The WA solution was added into each well at different concentration (1, 5, 10, 15, 20, and 25 µM/mL), and the plates were incubated for 24 h in a CO_2_ incubator at 37 °C. After incubation, 10 µL/mL MTT solutions (5 mg/mL stock) were added into each well, followed by incubation for 4 h at 37 °C. A similar set of experiment was carried out for the vehicle-treated group. Formazan crystals were solubilized with 100 μL DMSO, and the absorbance was measured at 590 nm using a microplate reader (BioTek Instruments, Inc., Santa Clara, CA, USA). The percentage cell proliferation inhibition potential was calculated as per a formula described elsewhere [20]. The 50% inhibitory concentration (IC_50_) for both the cell lines was calculated by GraphPad Prism 5.0 software using a regression analysis tool.

### 2.3. Small RNA Sample Preparation

Triple-negative breast cancer cells (MDA-MB-231) cells were treated with Withaferin A at IC_50_ concentration and DMSO (0.1%) for 24 h. DMSO-treated samples were considered as the vehicle control group. After 24 h incubation, the cells were harvested, and small RNAs were isolated using the mirVana^TM^miRNA isolation kit (Catalog# P/N 15604, Invitrogen, Thermo Fisher Scientific, Waltham, MA, USA) according to the manufacturer’s instructions. A Dnase treatment was given to samples to exclude the genomic DNA contamination using a Qiagen kit (Cat: 79254). RNA concentration and purity were estimated using a Nanodrop200 spectrophotometer (Thermo Fisher Scientific, USA) and further validated by a Qubit 2.0 Fluorometer (Thermo Fisher Scientific, USA). The RNA integrity was determined using a 2100 Bioanalyzer (Agilent 2100 expert) and checked by an Agilent 2100 Bioanalyzer using a RNA 6000 Pico Kit (Agilent Technologies, Santa Clara, CA, USA) [21]. 

### 2.4. Library Construction and Small RNA Next Generation Sequencing

A small RNA sequencing library was prepared for the vehicle- and Withaferin-A-treated groups using the NEXTflex™ Small RNA-Seq Kit (Cat: 513206). A total of 1000 ng RNA from each sample was used for small RNA library preparation. Adapter-ligated fragments were reverse transcribed with M-MuLV Reverse transcriptase by priming with reverse transcription primers. The Illumina-compatible cDNA libraries were quantified using a Qubit dsDNA HS assay kit (Invitrogen, Cat: Q32854) by Qubit fluorometer (Thermo Fisher Scientific MA, USA). The sequencing was carried out using 75 cycles SE chemistry on an Illumina NextSeq 550 High Output platform following the manufacturer’s protocol to generate raw reads, which were further subjected to trim the adopters, followed by length filtering. Bowtie-1.1.1 was used to align the sequenced reads against the GRCh38.p10 genome. Contaminated reads, those matching other ncRNAs (r, t, sn, and snoRNAs), were removed. High quality > q30 and non-redundant reads were considered as clean reads that were used for miRNA prediction and count profile generation. Homology and non-gapped alignment approaches were used for known miRNA prediction against human-matured miRNA sequences in miRbase V.22 using ncbi-blast-2.2.30 [22,23]. The data discussed in this manuscript were deposited in NCBI’s Gene Expression Omnibus and are accessible through GEO accession number GSE183395.

### 2.5. Differential Expression (DE) Analysis

Differential expression analysis of miRNAs in Withaferin-A-treated MDA-MB-231 cells compared to vehicle-treated cells was performed in triplicate using the DESeq2 tool. Normalization of the variations in the clean reads was done by using the library normalization method selected from the DESeq library. To assess the differential expression, a log2 fold value of 1.5 was used as cutoff. The miRNAs with log2 fold > 1.5 were considered as “UP” regulated; miRNAs with log2 fold < −1.5 were considered as “DOWN” regulated. The volcano plots for differentially expressed miRNAs (DEMs) were generated using GraphPad prism software 5 [21].

### 2.6. Microrna Target Gene Prediction and Functional Enrichment Analysis

The target genes of significantly expressed DEMs were predicted using the online miRSystem database. The miRSystem tool is an integration of seven well-known gene prediction algorithms (DIANA, miRanda, miRBridge, PicTar, PITA, rna22, and TargetScan) and two validated databases (Tarbase and miRecords). The target genes that followed hit > 3 were sorted and used for functional analysis. The predicted target genes were submitted to the online Database for Annotation, Visualization, and Integrated Discovery (DAVID) program for Gene ontology (GO) and Kyoto Encyclopedia of Genes and Genomes (KEGG) pathways analysis. Only the significant (*p* < 0.05) GO term and KEGG pathways were considered [21].

### 2.7. Quantitative RT-PCR Validation of Top 5 up and Top 5 down DEMs 

The top 5 up and top 5 down DEMs (from NGS results) were subjected to validation using the SYBR green-based qRT-PCR technique in Withaferin-A-treated MDA-MB-231 and MDA-MB-453 cell lines. Both the cell lines were treated with Withaferin A at IC_50_ concentration, and miRNA was isolated from the experimental and control groups using a mirVana^TM^ miRNA isolation kit (Catalog# P/N 15604, Invitrogen, Thermo Fisher Scientific, Waltham, MA, USA) according to the manufacturer’s instructions. The cDNA was synthesized by using a first strand cDNA synthesis kit (Takara Catalog No. 683183) as per manufacturer protocol. In brief, small RNAs were isolated from test samples of each group using the mirVana isolation kit (Invitrogen Thermo Fisher Scientific, Waltham, MA, USA, Catalog No. P/N 15604) and were subjected to quantity/quality check using a Nano Drop (ND)-1000 spectrophotometer (Thermo Fisher Scientific, Walthem, MA, USA). A total of 500 ng miRNA was polyadenylated by poly (A) polymerase enzyme and reverse transcribed into cDNA by reverse transcriptase enzyme using an miRNA first strand cDNA synthesis kit (Takara Catalog No. 683183). The reaction mixture was incubated at 37 °C for 1 h in the PCR machine followed by enzyme inactivation at 85 °C up to 5 min. qRT-PCR was performed using a universal SYBR green master mix 2X with miRNAs-specific 5′ forward primer and 3′ universal backward primers provided by the manufacturer with the first strand cDNA synthesis kit (Takara Cat: 683183). Each reaction was performed in a final volume of 12.5 μL containing 1 µL of 10X diluted cDNA, 6.25 µL of 2X SYBR green PCR master mix, 0.25 μL of each (5′ miRNA-specific and 3′ universal) primer, and 4.75 µL of nuclease free RT-PCR grade water. RT-PCR was run with an initializing step at 95 °C for 3 min, denaturation at 95 °C for 10 s, annealing at 60 °C for 30 s, and extension at 72 °C for 30 s and repeated for 40 cycles using Quantstudio^®^ 3, RT-PCR Applied Biosystem, Thermo Fischer Scientific, USA. For normalization, expression of the RNAU6 was used as an internal control [21]. The miRNA fold-change expression was calculated by using the 2^−∆∆^ CT method. The list of designed primers of miRNAs and their respective sequences are provided in Supplementary Table S1.

### 2.8. Transient Transfection of miR-181c-5p Mimic 

The TNBC cells were transiently transfecting with a miR-181c-5p mimic (mature miRNA sequence: AAC AUU CAA CCU GUC GGU GAG U) (Catalog: 4464066 and Assay ID: MC10181; Ambion, Thermo Fisher Scientific, Waltham, MA, USA) using the Lipofectamine^®^ 3000 (Catalog: L3000008, Invitrogen, Thermo Fisher Scientific, Waltham, MA, USA) transfection reagent. The miRNA mimic scrambled sequence (Catalog: 4464058; Ambion; Thermo Fisher Scientific, Waltham, MA, USA) transfected TNBC cells were considered as negative control. The final concentration of the miRNA mimic and negative control was 60 nm. The cells were transfected for 24, 48, and 72 h. To assess the transfection efficacy, the effect (gain of function) of miRNA mimic transfection was measured compared to negative control group [20]. For this, the total miRNA was isolated using a mirVanaTM miRNA isolation kit (Catalog# P/N 15604, Invitrogen, Thermo Fisher Scientific, Waltham, MA, USA) and a SYBER green-based qRT-PCR analysis was performed as per the methodology discussed in Section 2.8.

### 2.9. Effect of Withaferin A and miR-181c-5p Mimic Co-Treatment on TNBC Cell Viability

The TNBC cell lines, MDA-MB-231 and MDA-MB-435, were seeded at the density of 6000 cells/well in 96-well plate and incubated overnight as per the methodology discussed in the material and methods section under the heading of cell lines. Then, the incubation cells were transfected with the miRNA mimic negative control (Catalog: 4464058) and miR-181c-5p mimic (Catalog: 4464066 and Assay ID: MC10181) for 24, 48, and 72 h at a 60 nm final concentration. Furthermore, the effect of WA (IC_50_) and miRNA mimic co-treatment in 24 h exposure was also studied in TNBC cells. The cell viability assay was performed as per the methodology discussed in the material and methods section under the heading of cell cytotoxicity assay.

### 2.10. Confocal Microscopy

Triple-negative breast cancer cells (MDA-MB-231) cells (2 × 10^6^) were seeded into each well of a 6-well plate and left overnight to adhere; afterwards, cells were transfected with the miR-181c-5p mimic alone or co-treatment with WA (IC_30_ and IC_50_) for 24 h. After incubation, the cells were harvested and immediately stained with the Hoechst 33342 dye (5 µg/mL) to study the morphological nuclear changes [12]. Furthermore, the cells were separately stained with the JC-1 dye (5 μM/mL) to check the mitochondria membrane potential (ΔΨm) in test cells [12]. Moreover, 2, 7-dichlorodihydrofluorescein diacetate (H2DCFDA) dye mediated staining (1 µM/mL) of the cells was performed to assess the reactive oxygen species (ROS) generation [12]. The stained cells for each individual experiment were kept at room temperature for 20 min and 4 h in a dark place. After incubation, the cells were washed thrice with PBS, and without delay, the stained cells were examined using a confocal laser scanning microscope (CLSM) facility available in the Central Instrumentation Laboratory (CIL) of the University. The results were compared with vehicle treated cells.

### 2.11. Annexin V/FITC and PI Apoptosis Assay

MDA-MB-231cells were cultured in a 6-well plate at a density of 1 × 10^5^ cells/well. After 24 h incubation, cells were transfected with miR-181c-5p mimic alone or co-treatment with WA (IC_30_ and IC_50_) for 24 h. After that, samples were washed with PBS and diluted with annexin V binding buffer. Then, 5 μL of Annexin V—FITC conjugate and 5 μL of PI Solution were added to the samples and incubated for 15 min at room temperature with protection from light. The cells were observed under confocal microscopy (Olympus Fluoview).

### 2.12. Expression Analysis of Cell Cycle and Apoptosis Related Genes

The expression levels of cell cycle genes (CDK4 and cyclin D1) and proapoptotic/apoptosis initiator (Caspase 8, 3 and BAX) and anti-apoptotic (PARP and BCL2) marker genes were estimated using qRT-PCR technique in test samples. The total RNA was isolated from the vehicle- and WA- (IC_30_ and IC_50_) treated; miRNA mimic and miRNA-mimic negative control transfected; and miRNA mimic co-treated with WA (IC_30_ and IC_50_) TNBC cells in 24 h exposure. The cDNA was synthesized from the isolated RNA (1 µg) of each group using iScript cDNA synthesis kit (Cat. #1708891). The qRT-PCR expression analysis was performed by adding 1 µL of cDNA, 2.5 uL of 2X SYBR green PCR master mix, 0.1 μL of each (forward and reverse) primer, and 6.3 µL of nuclease free RT-PCR grade water. The qRT-PCR was run with an initializing step at 95 °C for 3 min, denaturation at 95 °C for 15 s, annealing at 55 °C for 60 s, and extension at 72 °C for 60 s and repeated for 40 cycles using Quantstudio^®^ 3, RT-PCR Applied Biosystem, Thermo Fischer Scientific, USA. GAPDH was used as the endogenous control to normalize the expression level. The cycle of threshold (Ct) value of each sample was calculated by using the 2^−∆∆^ CT method for the fold-change expression of mRNA expression [12,20,24]. The primer sequence of test genes (caspase 3, caspase 8, BAX, PAPR, Bcl2, and GAPDH) is provided in Supplementary Table S2.

### 2.13. Caspase-Glo^®^ 3/7 Activity 

Cell apoptosis detection was done using the Caspase-Glo^®^ 3/7 assay kit (Catalog: G8090). For this, 20000 MDA-MB-231 cells/well were seeded in the 96-well plate and incubated overnight. After that, cells were transfected with miR-181c-5p mimic, miRNA mimic negative control, and cells were co-transfected with mimic and withaferin A (at IC_30_ and IC_50_) for 24 h and incubated at 37 °C in 5% humidified CO_2_ incubator. Then, 100 µL of Caspase-Glo^®^ 3/7 reagent was added into each well and incubated up to 2 h. After incubation periods, Caspase 3/7 activity was measured by taken luminescence reading using Filter Max Pro 5 Multi-Mode Microplate Reader (Molecular Devices, San Jose, CA, USA); results are reported as relative light units (RLU).

### 2.14. Western Blotting Analysis of Cell Cycle and Apoptotic Proteins 

The Western blot technique was used to determine the expression levels of apoptotic proteins during the apoptosis event in mimic transfected, mimic scrambled oligonucleotides transfected, co-transfected with mimic and two different concentration of Withaferin A (IC_30_ and IC_50_), vehicle-treated and Withaferin A (IC_30_ and IC_50_)-treated MDA-MB-231 cells. Cells were seeded at the density of 2 × 10^5^ cells/well in a 6-well plate and left overnight to adhere with the surface. Then, cells were transfected, co-transfected, and treated and incubated for 24 h. Then, cells were harvested and lysed in RIPA buffer to isolate the proteins, followed by 30 min incubation on ice, and centrifuged at 13,000 RPM for 15 min. The Bradford method was used to estimate the isolated protein. After that, 50 µg/lane protein was loaded into each well and protein bands were resolved on 12% SDS-PAGE. The resolved protein bands were transferred on PVDF membrane. The PVDF membrane was incubated with antibodies of CDK4 with 2 µg/mL (Catalog: PAB233Hu01, Cloud-Clone-Corp, Katy, USA), cyclin D1 with 1:500× dilution (Catalog: MA514512, Invitrogen, Thermo Fisher Scientific, Waltham, MA, USA), anti-caspase 3 with 1:1000× dilution (Catalog: ab32351, Abcam, Cambridge, UK), caspase 8 with 2 µg/mL concentration (Catalog: PA520118, Invitrogen, Thermo Fisher Scientific, Waltham, MA, USA), BAX with 1:1000× dilution (Catalog: MA532031, Invitrogen, Thermo Fisher Scientific, USA), and β-actin with 1:1000× dilution (Catalog: MA532540, Invitrogen, Thermo Fisher Scientific, Waltham, MA, USA) at 4 °C overnight, followed by blocking with 5% non-fatted milk. After membrane washing with TBST buffer (pH-7.2), the membrane was incubated with horseradish peroxidase-conjugated secondary antibody with a 1:2000× dilution for 2 h at room temperature followed by three washes with TBST for 15 min (each wash at 5 min interval). The bands were developed with the enhanced chemiluminescence (ECL) system (Thermo Fisher Scientific). The signals were captured through the ChemiDoc imaging instrument (Bio-Rad), Hudson MA, USA and the image was processed using image lab software 6.0.1 Bio-Rad, Hudson MA, USA [12,20].

### 2.15. Statistical Analysis

The data are shown as mean ± SD at a 95% confidence interval level over three independent experiments. The results were statistically significant at a probability level of *p*-value < 0.05. The statistical analysis was performed using the Graphpad prism software. Two-tailed Student’s t-tests were performed for the comparison between the groups.

## 3. Results

### 3.1. Withaferin A Induces Cytotoxicity in Triple-Negative Breast Cancer Cells

**Figure 1:**
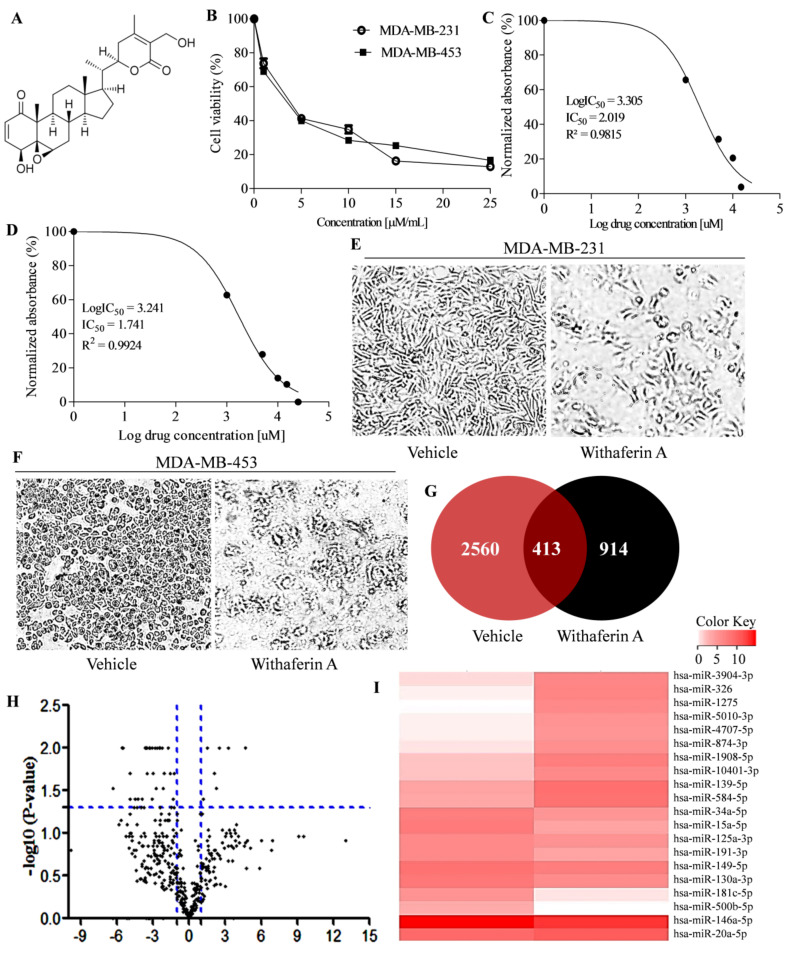
The effect of Withaferin A on the miRNA expression profile in triple-negative cancer cells. (**A**) Chemical structure of Withaferin A adapted from PubChem (PCID 265237) (**B**) Effect of Withaferin A on MDA-MB-231 and MDA-MB-453 cells in 24 h treatment (**C**,**D**) Calculation of IC_50_ concentration in 24h Withaferin A treatment in MDA-MB-231 and MDA-MB-453 cells using regression analysis, respectively. (**E**) Effect of vehicle and Withaferin A treatment on MDA-MB-231 and (**F**) MDA-MB-453 cells morphology at IC_50_ concentration in 24h treatment, respectively. (**G**) Venn diagram analysis showing common miRNAs expressed in vehicle- and Withaferin A (IC_50_)-treated MDA-MB-231 cells in 24h exposure. (**H**) Volcano plot showing significant differential expression of miRNAs in Withaferin A (IC_50_)-treated MDA-MB-231 cells in 24h exposure compared to vehicle-treated cells. (**I**) Heatmap showing expression pattern of top twenty differentially expressed miRNAs in Withaferin A (IC_50_)- and vehicle-treated group in 24 h exposure.

An MTT assay was utilized to assess the cytotoxic effect of Withaferin A (WA) in triple-negative breast cancer cells (Figure 1A). In the present study, we utilized MDA-MB-231 and MDA-MB-453 cell lines as in vitro TNBC models. The test cells were treated at different concentrations (1–25 µM) of WA for 24 h. A concentration-dependent decrease in cell viability was observed in TNBC cells in 24 h treatment (Figure 1B). Furthermore, we utilized the cell viability results at different concentrations and calculated a 50% inhibitory concentration (IC_50_) of WA for MDA-MB-231 and MDA-MB-453 cells, which was ~2 and ~1.7 µM respectively (Figure 1C,D). Moreover, we studied the effect of WA IC_50_ concentration on cytotoxic and morphological effects in MDA-MB-231 and MDA-MB-453 cells in a 24 h treatment. The microscopic evaluation of vehicle- (0.1% DMSO) and WA-IC_50_-concentration-treated TNBC cells revealed changes in the cellular morphology of TNBC cells (Figure 1E,F).

### 3.2. Withaferin A Treatment Altered MiRNA Expression Profile in MDA-MB-231 Cells

Although WA is well reported for its cell proliferation inhibition and apoptosis induction in breast cancer cells, its potential to alter the miRNA expression in cancer cells has not yet been studied. To explore the underlying mechanisms of WA, we utilized high-throughput small RNA sequencing to identify the changes in miRNA expression of MDA-MB-231 cells after WA IC_50_ treatment in 24 h. The results were compared to vehicle-treated cells. The independent three replicates of each group were sequenced. The sequenced miRNA reads obtained in WA- (n = 3) and vehicle-treated (n = 3) MDA-MB-231 cells revealed a total of 2973 and 1327 known miRNAs expressed in the vehicle- and WA-treated groups, respectively. Furthermore, we performed Venn diagram analysis to find the number of miRNAs present in both the groups. A total of 413 miRNAs expressed in both vehicle- and WA-treated MDA-MB-231 cells were considered as differentially expressed miRNAs (DEMs) (Figure 1G). A list of 413 DEMs among the test groups are provided in Supplementary Table S3. A total of 93 up-expressed and 188 down-expressed miRNAs were found in the WA-treated group compared to the vehicle-treated cells. Moreover, to discover the significant (*p* < 0.05) DEMs, the criteria log2FC)| ≥ 1.5 (up-expressed miRNAs) and FC| ≤ −1.5 (down-expressed miRNAs) were used. A total of 53 significant (*p* < 0.05) DEMs were identified in WA-treated MDA-MB-231 cells, and they are visualized in Figure 1H in the form of volcano plot. The expression pattern of the top 20 most differentially expressed miRNAs is depicted in the heatmap (Figure 1I). miR-181c-5p, miR-15a-5p, miR-500b-5p, miR-191-3p, and miR-34a-5p were the top five up-expressed DEMs and miR-1275, miR-326, miR-1908-5p, miR-3940-3p, and miR-139-5p were the top five down-expressed DEMs in WA-treated cells compared to the vehicle-treated group, respectively. The Log2FC and *p* values of the top five up and down-expressed miRNAs are listed in Supplementary Table S4.

### 3.3. Target Gene Prediction and GO Enrichment Analysis

**Figure 2:**
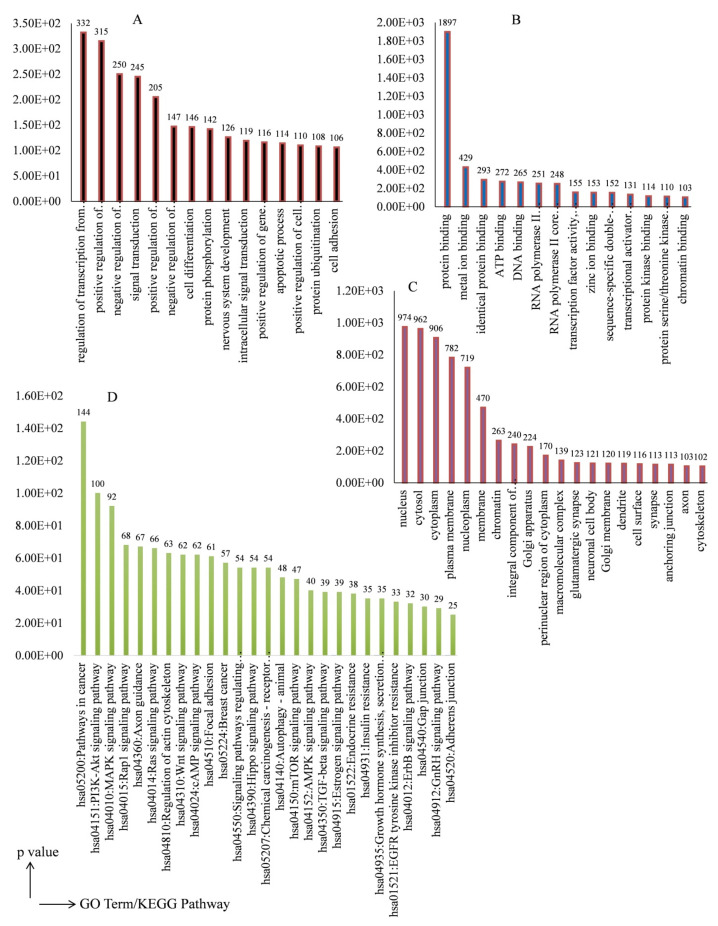
Gene ontology and KEGG pathway analysis of target genes of differentially expressed miRNAs in Withaferin-A-treated MDA-MB-231 cells. Differentially expressed miRNAs target gene enrichment in different GO terms (**A**) Biological process (**B**) Molecular function, and (**C**) Cellular component. The respective events of different GO terms were selected on the basis of significance value (*p* < 0.0001) and count number ≥ 100. Significant values of the events are mentioned above the respective term bar. (**D**) The significant (*p* < 0.0001) target genes of the differentially expressed miRNAs in MDA-MB-231 cells enriched in different KEGG pathways. Fold enrichment (gene count) values are shown above the respective pathway bar.

Next, to predict the biological role of the DEMs, we identified their target genes. The miRsystem-based analysis revealed a total of 4042 (Hit > 3) target genes for significant differentially expressed miRNAs, which are provided in Supplementary Table S5. The identified genes (Hit > 3) were annotated with biological processes (BP), cellular components (CC), and molecular function (MF) gene ontologies. The target genes of differentially expressed miRNAs were significantly (*p* < 0.05) enriched in 1148 GO terms. The significant (*p* < 0.0001) GO terms (BP, CC, and MF) of the WA treatment (IC_50_) mediated significant DEMs with count number > 100 are depicted in Figure 2A–C. The positive regulation of transcription, signal transduction, and apoptosis was found as a major BP in GO term analysis. Similarly, cytoplasm, plasma membrane, cell junctions, protein binding, DNA binding, and Kinases activity were found as important CC and MF in GO term analysis.

### 3.4. KEGG Pathway Enrichment Analysis

A KEGG pathway analysis for the identified target genes of the up- and down-expressed miRNAs was performed separately. The analysis showed that the predicted target genes of DEMs were significantly (*p* < 0.05) enriched in 144 signaling pathways. Next, we questioned whether the identified target genes are related to apoptosis-related pathways or not. For this, the enrichment of the target genes in KEGG pathway(s) related to apoptosis was studied. The results revealed that WA treatment modulated the DEMs target genes that were significantly (*p* < 0.05) enriched in apoptosis-related pathways (Figure 2D). Results showed that identified genes of significant DEMs were enriched in those signaling pathways that are actively involved in TNBC initiation and progression.

### 3.5. Withaferin A Potentially Induces Expression of miRNA-181c-5p in TNBC Cells

**Figure 3:**
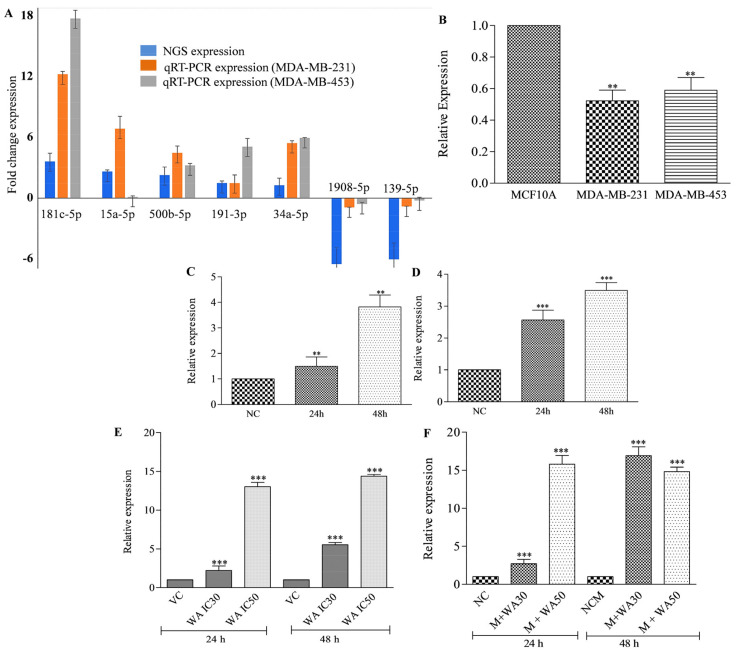
qRT-PCR expression analysis of differentially expressed miRNA(s). (**A**) Expression analysis of miRNAs in MDA-MB-231 and MDA-MB-453 cells using qRT-PCR technique compared with the next generation sequencing expression pattern. (**B**) Expression analysis of top differentially expressed miRNA (miR-181c-5p) in breast normal cells (MCF10A) and triple-negative breast cancer cells in a 24 h culture. (**C**) Expression analysis of miR-181c-5p in transient miR-181c miRNA transfected MDA-MB-231 cells in 24 h and 48 h exposure. (**D**) Expression analysis of miR-181c-5p in transient miR-181c miRNA transfected MDA-MB-453 cells in 24 h and 48 h exposure. The expression patterns of the miRNA in transfected cells were compared with the mimic negative control transfected cells. (**E**) Effect of Withaferin A treatment alone on miR-181c-5p expression in MDA-MB-231 cells in 24 h and 48 h exposure. (**F**) Effect of Withaferin A treatment in combination with miR-181c mimic on miR-181c-5p expression in MDA-MB-231 cells in 24 h and 48 h exposure. The qRT-PCR experiments were performed in triplicate independently and data are presented as mean ± standard error mean (SEM). Given values across the treatment are significantly different from each other at ** *p* < 0.05, where *** (*p* < 0.001).NC—negative control; M—miR-181c mimic; NCM—negative control mimic; WA30—Withaferin IC_30_; WA50—Withaferin IC_50_.

Next, to validate the NGS sequencing data, we investigated the expression pattern of the top five up- and down-expressed miRNAs in WA-treated (IC_50_) MDA-MB-231 cells. The miRNA expression of the 10 miRNAs was examined in WA-treated (IC_50_) MDA-MB-231 and MDA-MB-453 cells in 24 treatment using the qRT-PCR technique. hsa-miR-181c-5p, hsa-miR-15a-5p, hsa-miR-34a-5p, hsa-miR-500b-5p, hsa-miR-191-3p, hsa-miR-139-5p, and hsa-miR-1908-5p were successfully validated in the qRT-PCR experiment (Figure 3A). The NGS and qRT-PCR results showed that miR-181c-5p was highly differentially expressed in WA-treated TNBC cells among all the validated DEMs (Figure 3A). Therefore, miR-181c-5p was considered the lead miRNA for further experiments. In this sequence, we investigated the expression pattern of miR-181c in breast normal cells (MCF-10A). The results showed that miR-181c was significantly down-expressed in MDA-MB-231 and MDA-MB-453 cells compared to breast normal cells (Figure 3B). Next, we studied the miR-181c mimic transfection efficacy in MDA-MB-231 and MDA-MB-453 cells. For this, the time dependent expression pattern of miR-181c was studied in mimic transfected TNBC cells. The results revealed 1.5- and 3.8- fold up-expression of miR-181c in mimic treated MDA-MB-231 cells after 24 and 48 h of the transfection, respectively (Figure 3C). Similarly, 2.5- and 3.6-fold up-expression of miR-181c was noticed in 24 and 48 h mimic transfected MDA-MB-453 cells, respectively (Figure 3D).

To crosscheck the WA-treatment-mediated up-expression of miR-181c in TNBC cells, we studied the miRNA expression in WA alone and mimic transfected MDA-MB-231 cells co-treated with the different WA concentrations (IC_30_ and IC_50_) at different time (24 h and 48 h) intervals (Figure 3E,F). The findings of the experiment revealed a significant increase in miR-181c expression in the mimic and WA co-treated MDA-MB-231 cells in concentration- and time-dependent manners compared to mimic negative control transfected cells (Figure 3E). The expression of miR-181c was 2.7- and 15.8-fold higher in co-treated MDA-MB-231 cells after 24 h of the treatment, respectively. Similarly, in the 48 h co-treatment of MDA-MB-231 cells, 16.9- and 14.8-fold up-expression was determined (Figure 3E).

### 3.6. Withaferin A and miR-181c-5p Mimic Co-Treatment Decreases Cell Proliferation in TNBC Cells

**Figure 4:**
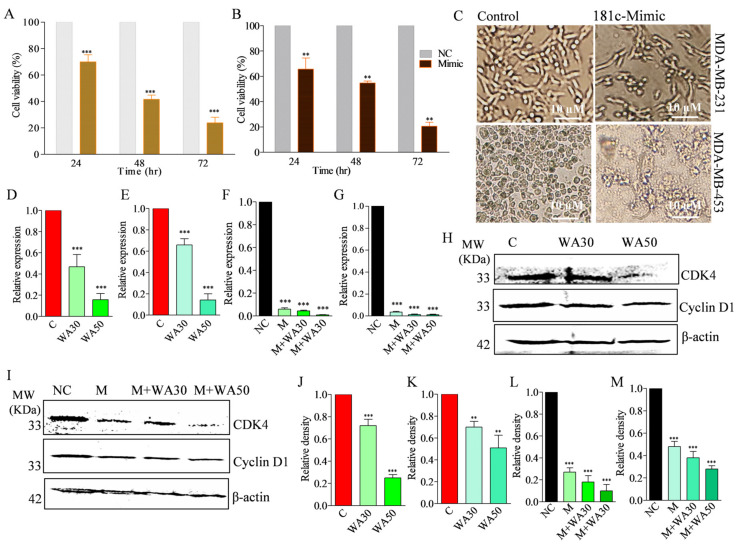
The effect of miR-181c-5p forced expression on cell viability and morphology, and the effect of Withaferin A and co-treatment (mimic and Withaferin) on cell-cycle marker proteins in triple-negative breast cancer cells. (**A**) Effect of miR-181c-5p forced expression on MDA-MB-231 cell viability in 24, 48, and 72 h exposure at 60 nm concentrations. (**B**) Effect of miR-181c-5p forced expression on MDA-MB-453 cell viability in 24, 48, and 72 h exposure at 60 nm concentrations. (**C**) Effect of miR-181c-5p forced expression on MDA-MB-231 and MDA-MB-453 cell morphology in 24 h exposure at 60 nm concentration. The mRNA gene expression of (**D**) CDK4 and (**E**) CCND1 in Withaferin A (IC_30_ and IC_50_)-treated MDA-MB-231 cells in 24 h exposure compared to the vehicle control. (**F**) CDK4 and (**G**) CCND1 mRNA expression in miR-181c-5p mimic transfected and co-treated (mimic + WAIC30 and mimic + WAIC50) MDA-MB-231 cells in 24 h exposure in comparison to negative control. (**H**) Protein expression level of CDK4 and cyclin D1 in Withaferin A (IC_30_ and IC_50_) 24 h treated MDA-MB-231 cells. (**I**) Protein expression level of CDK4 and cyclin D1 in miR-181c-5p mimic transfected and co-treated (mimic + WAIC30 and mimic + WAIC50) in 24 h incubated MDA-MB-231 cells. Relative densities of immuno bands of (**J**) CDK4, (**K**) cyclin D1 in Withaferin A (IC_30_ and IC_50_) 24 h treated MDA-MB-231 cells; (**L**) CDK4 and (**M**) cyclin D1 in miR-181c-5p mimic transfected and co-treated (mimic + WAIC30 and mimic + WAIC50) in 24 h incubated MDA-MB-231 cells, estimated by image lab software 6.0.1 Bio-Rad. Cell viability, qRT-PCR, and Western blot experiment data are representative of three independent experiments. *** *p* < 0.001 (ANOVA); ** *p* < 0.01 (ANOVA) compared with respective control. Values represent mean ± standard error mean (SEM) (n = 3).

As we found a significantly lower expression of miR-181c in TNBC cells compared to normal breast cells, we studied the effect of miR-181c forced expression on the cell viability of MDA-MB-231 and MDA-MB-453 cells. For this, the TNBC cells were treated with the miR-181c mimic for different time intervals and the cell viability was measured using an MTT assay. Results revealed that up-expression of miR-181c suppressed cell proliferation up to 30.08, 58.43, and 76.21% in 24, 48, and 72 h transfection, respectively, in MDA-MB-231 cells (Figure 4A). Similarly, a decrease of 34.41, 45.35, and 79.56 % cell viability in 24, 48, and 72 h transfection, respectively, was measured in MDA-MB-453 cells (Figure 4B). The negative control mimic transfected TNBC cells were used to compare the results. Furthermore, we studied the effect of forced miR-181c expression on alterations in TNBC cell morphology in 24 h treatment. The results showed that in 24 h treatment, the morphology of MDA-MB-231 and MDA-MB-453 cells was changed compared to negative control mimic transfected cells (Figure 4C). Moreover, to examine the effect of WA-treatment-mediated up-expressed miR-181c on the cell cycle, we studied cell cycle G1 phase markers (CDK4 and cyclin D1) in MDA-MB-231 cells in the presence of the miRNA mimic in 24 h treatment. The findings of the experiment revealed a significant decrease in CDK4 and cyclin D1 expression at the mRNA and protein level (Figure 4D–I).

### 3.7. Withaferin A and miR-181c-5p Mimic Co-Treatment Potentiates Nuclear Morphology Alterations, Mitochondria Membrane Potential Decrease, Reactive Oxygen Species Generation, and Apoptotic Cell Population in MDA-MB-231 Cells

**Figure 5:**
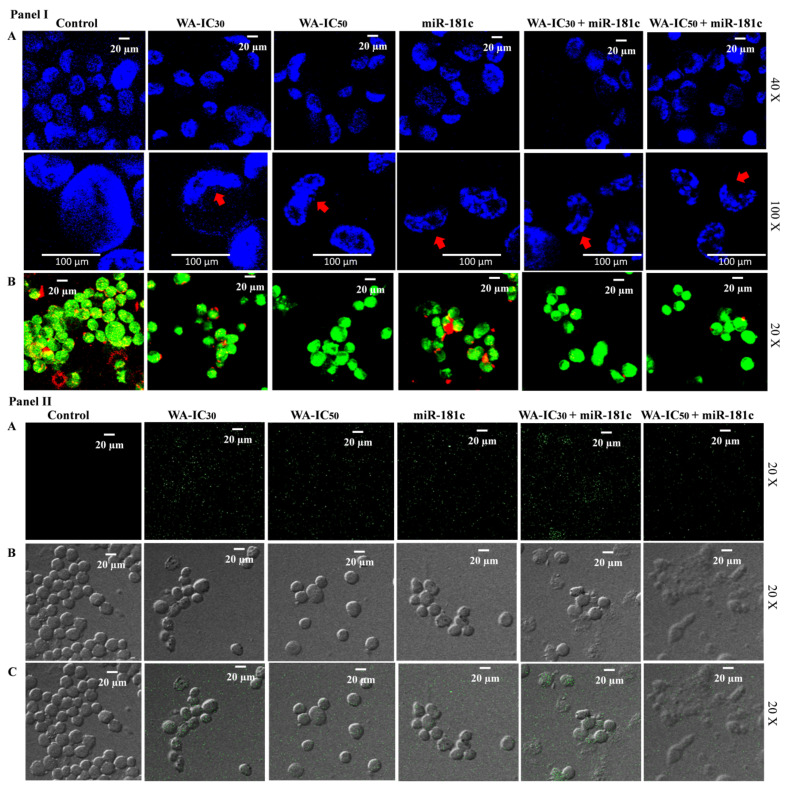
The effect of miR-181c-5p mimic and Withaferin A co-treatment on apoptosis-related morphological changes, mitochondrial membrane potential, and reactive oxygen species generation in triple-negative breast cancer cells. Panel I: (**A**) Effect of Withaferin A and miR-181c-5p alone and in co-treatment on apoptosis-related morphological changes in MDA-MB-231 cell viability in 24 h exposure. Panel I: (**B**) Effect of Withaferin A and miR-181c-5p alone and in co-treatment on mitochondrial membrane potential in MDA-MB-231 cell viability in 24 h exposure. Panel II: Effect of Withaferin A and miR-181c-5p alone and in co-treatment on reactive oxygen species generation in MDA-MB-231 cell viability in 24 h exposure. (**A**–**C**). The results were compared with the control (vehicle-treated) group.

Morphological change in the cancer-cell nucleus is a well-established phenomenon studied to assess the apoptosis induction potential of anticancer compounds. Using Hoechst 33342, we tested whether WA-induced miR-181c expression has the potential to initiate apoptosis-related nuclear morphological changes in TNBC cells or not. Results showed that WA treatment increased concentration-dependent morphological changes in MDA-MB-231 cells in 24 h exposure. Moreover, the forced expression of miR-181c in test cells also induced the morphological changes in test cells in 24 h exposure. Interestingly, co-treatment of miRNA-181c mimic with the increased concentration of WA (IC_30_ and IC_50_) showed better nuclear morphological changes in MDA-MB-231 cells in 24 h exposure compared to the vehicle control and alone treatment group (Figure 5A panel I).

A decrease in mitochondrial membrane potential (ΔΨm) is implicated in apoptotic induction in cancer cells after the treatment of anticancer phytochemical(s) and miRNA(s). Using JC-1, a cationic dye, we tested whether WA-induced miR-181c expression has the potential to decrease ΔΨm in TNBC cells or not. Intact mitochondria possess a negative charge, which allows the JC-1 entrance and formation of J-aggregates producing red fluorescence. The collapse of ΔΨm in apoptotic cells results in the accumulation of JC-1 dye in the cytoplasm, which produces a green color. Results showed that WA treatment increased concentration-dependent green fluorescence in MDA-MB-231 cells in 24 h exposure. The forced expression of miR-181c in test cells produced comparatively less green fluorescence than WA-treated cells in 24 h exposure. Interestingly, co-treatment of miRNA-181c mimic with the increased concentration in WA increased the green fluorescence in a concentration-dependent manner (Figure 5B panel I). 

ROS are implicated in the induction of apoptosis by a number of natural anticancer drugs and miRNA(s). Although the ROS generation potential of WA in cancer cells has been reported, we questioned whether WA-induced miR-181c expression is involved in the ROS-production-mediated proapoptotic response or not. Using H2DCFDA, a reduced fluorescein molecule that enters cells and, following acetate group cleavage, produces a green color fluorescence, we investigated this possibility. Results revealed that WA (at IC_30_ and IC_50_) treatment and miR-181c forced expression increased green fluorescence in MDA-MB-231 cells compared to vehicle treated cells (Figure 5 panel II). Interestingly, WA (IC_30_) and miR-181c mimic co-treatment produced more ROS (green fluorescence) compared to the alone treatment. Comparatively, WA (IC_50_) and miR-181c mimic co-treatment produced lesser ROS, but the cells were destroyed more in this group.

### 3.8. Withaferin A and miR-181c-5p Mimic Potentiates Caspase-Mediated Apoptosis Induction in MDA-MB-231 Cells

**Figure 6:**
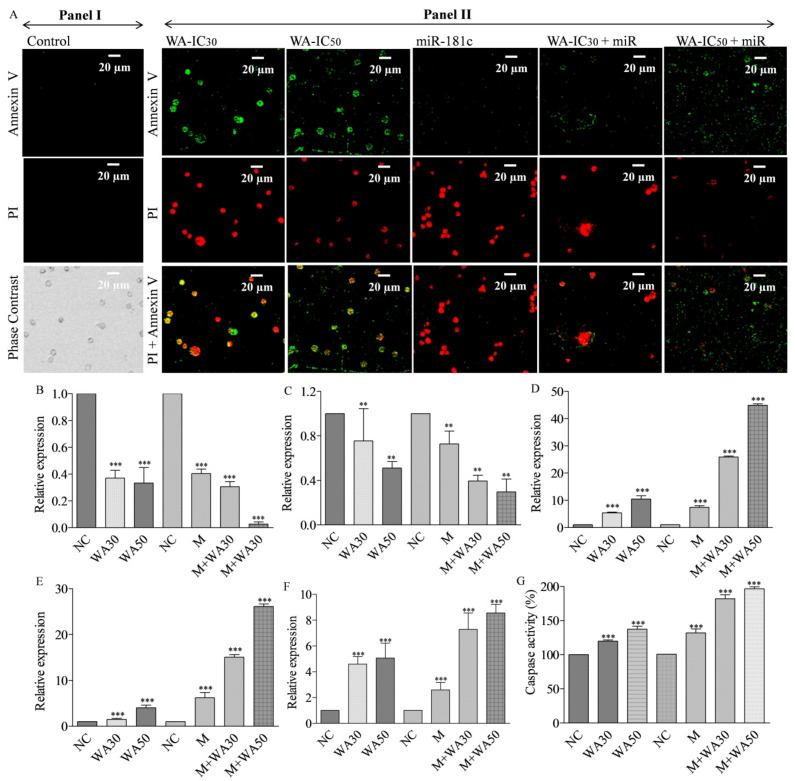
The effect of Withaferin A and mi-181c-5p mimic co-treatment on the apoptotic cell population, apoptosis-related marker expression, and caspase enzymatic activity in TNBC cells. (**A**) Panel I: Apoptosis event in miR-181c-5p mimic negative control transfected MDA-MB-231 cells detected by annexin V and PI. Panel II: Apoptosis induction in Withaferin A (IC_30_ and IC_50_), miR-181c-5p mimic and their co-treatment in MDA-MB-231 cells in 24 h exposure. qRT-PCR based expression analysis of apoptosis-related markers. (**B**) PARP, (**C**) BCL-XL, (**D**) Caspase 3, (**E**) Caspase 8 (**F**), and BAX in Withaferin A (IC_30_ and IC_50_), miR-181c-5p mimic, and their co-treatment in MDA-MB-231 cells in 24 h exposure. (**G**) Caspase 3/7 enzymatic activity measured by Glo caspase 3/7 assay kit in Withaferin A (IC_30_ and IC_50_), miR-181c-5p mimic, and their co-treatment in MDA-MB-231 cells in 24 h exposure. The data presented as mean ± SD. Experiments were performed in triplicate and represented by mean ± standard error mean (SEM). Expression values across the treatment are significantly different from each other at *p* < 0.05. ** (*p* < 0.01) and *** (*p* < 0.001).

**Figure 7:**
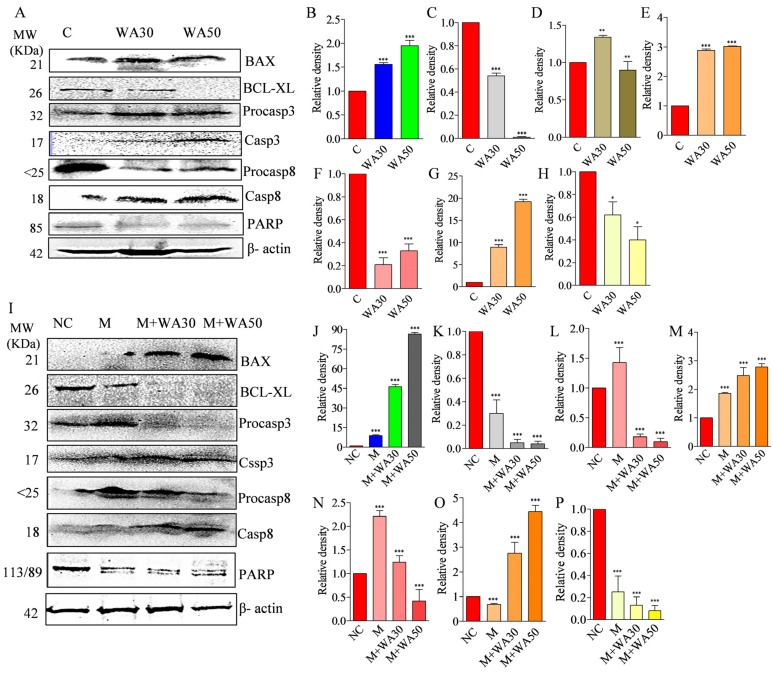
The effect of Withaferin A, miR-181c-5p mimic, and their co-treatment on apoptosis-related protein markers in triple-negative breast cancer cells. (**A**) Protein expression level of BAX; BCL-XL; Procaspase 3, 8; Cleaved caspase 3, 8; and PARP in WA IC_30_ and IC_50_ concentration treated MDA-MB-231 cells in 24 h. Relative densities of immuno bands of (**B**) BAX, (**C**) BCL-XL, (**D**) Procaspase 3, (**E**) Cleaved caspase 3, (**F**) Procaspase 8, (**G**) Cleaved caspase 8, and (**H**) PARP were estimated by image lab software 6.0.1 Bio-Rad. (**I**) Protein expression level of BAX; BCL-XL; Procaspase 3, 8; Cleaved caspase 3, 8; and PARP in miR-181c-5p mimic transfected and mimic + WA (IC_30_ and IC_50_) co-treated MDA-MB-231 cells in 24 h. Relative densities of the immuno bands of (**J**) BAX, (**K**) BCL-XL, (**L**) Procaspase 3, (**M**) Cleaved caspase 3, (**N**) Procaspase 8, (**O**) Cleaved caspase 8, and (**P**) PARP estimated by image lab software 6.0.1 Bio-Rad. Data are representative of three independent experiments. *** *p* < 0.001 (ANOVA); ** *p* < 0.01 (ANOVA); * *p* < 0.01 (ANOVA) compared with respective control. Values represent mean ± standard error mean (SEM) (n = 3). NC—negative control; M—miR-181c mimic; WA30—Withaferin IC_30_; WA50—Withaferin IC_50_.

The effect of WA and miR-181c-5p mimic alone and in combination on apoptosis cell population was studied using Annexin-PI-based confocal microscopy in MDA-MB-231 cells. The results showed that WA alone induced apoptosis cell population in a concentration-dependent manner. The late apoptosis cell population was decreased at higher test concentrations (Figure 6A). Interestingly, miR-181c-5p mimic increased the late apoptosis cell population compared to treatment with WA alone. WA co-treatment increased the apoptotic cell population in mimic co-treated cells in a concentration-dependent manner (Figure 6A). Increased caspase 3 and 8, along with BAX expression and decreased PARP and BCL-XL gene expression, were implicated in apoptosis induction in breast cancer cells [25]. We questioned whether WA-induced miR-181c expression induces apoptosis in TNBC cells by modulating the expression of these molecular markers or not. For this, the mRNA and protein expression of these markers were studied at the mRNA and protein level, and the results are shown in Figure 6 and Figure 7. The results showed that the miR-181c-mimic-mediated decrease in PARP expression was further significantly lowered (up to 25-fold) in WA–mimic co-treated MDA-MB-231 cells in a concentration-dependent manner (Figure 6B). Similarly, a decreased expression of BCL-XL mRNA was observed in WA–mimic co-treated MDA-MB-231 cells compared to control cells (Figure 6C). Furthermore, the caspase 3, caspase 8, and BAX mRNA levels were significantly increased in a concentration-dependent manner in mimic–WA co-treated TNBC cells compared to non-treated and individual treatment(s) (Figure 6D–F). Despite having structural similarities, the effector caspase 3 and 7 have differing anticancer functions. In contrast to caspase 7, which seems to be more crucial to the loss of cellular viability, caspase 3 regulates DNA fragmentation and the morphological alterations of apoptosis. The coordinated function of both caspase is essential for the triggering of apoptosis in cancer cells [26]. Keeping these facts in our mind, we measured the effect of WA, miR-181c mimic, and their co-treatment on caspase-3/-7 activity in TNBC cells. For this, we utilized a Glo-caspase-3/7-based apoptosis detection kit. The results showed 19.8 and 37.3% increased caspase 3/7 activity in MDA-MB-231 cells at WA IC_30_ and IC_50_ concentrations, respectively, in 24 h treatment compared to vehicle-treated cells. Similarly, miR-181c forced expression increased the caspase 3/7 activity by 31.9 % compared to the mimic negative control. Interestingly, co-treatment of miR-181c with WA IC_30_ and IC_50_ concentration increased caspase 3/7 activity by 81.91 and 96.5 %, respectively, compared to mimic negative control transfected TNBC cells in 24 h (Figure 6D–G). Moreover, immunoblotting experiments were performed in WA IC_30_ and IC_50_ treated TNBC cells, as well as miR-181c-5p mimic alone and in combination with WA (IC_30_ and IC_50_) to study the apoptosis induction mechanism. The effect of WA (IC_30_ and IC_50_) and mimic–WA (IC_30_ and IC_50_) treatment on the expression levels of apoptosis pathway proteins are shown in Figure 7. Results showed that WA treatment increased the BAX/BCL-XL ratio in TNBC cells in a concentration-dependent manner (Figure 7B,C). WA treatment increased the active caspase 3 and caspase 8 protein levels in a concentration dependent manner (Figure 7D–G). Furthermore, the treatment decreased the levels of cleaved PARP enzyme in a concentration-dependent manner (Figure 7H). The combined effect of miR-181c-5p and WA treatment (IC_30_ and IC_50_) on apoptosis pathway proteins in TNBC cells in 24 h exposure is shown in Figure 7I. The results showed that co-treatment (mimic + WA IC_30_ and mimic + WA IC_50_) augmented the apoptosis pathway protein expression modulation potential of WA in test cells compared to treatment with WA alone (Figure 7J–P).

## 4. Discussion

The clinical management of triple-negative breast cancer is challenging due to its strong association with aggressiveness, therapy resistance, high metastasis potential, and early cancer relapse [12]. Additionally, the severe side effects, caused by the toxicity of the available chemotherapy, are also associated with poor management of the disease. Currently, natural anticancer agents are the preferred choice due to their lower side effects. WA is known to exert its effect on almost every hallmark of cancer at micro molar concentrations. Various studies reported the potent anticancer potential of WA in different in vitro and in vivo cancer experimental models [27]. Recently, it was reported that WA significantly decreased aggressiveness and metastasis potential in triple-negative breast cancer cells at test concentrations [17,18]. In the present study, we found that WA significantly decreased the cell viability of TNBC cells at micro molar concentrations (Figure 1A–F). Similarly, a different study also reported micro molar IC_50_ concentration against TNBC cells. This indicates the authenticity of our experimental data. To the best of our knowledge, to date, there has been no report available on the miRNA expression modulatory potential of WA in cancer or any other disease. In the present work, for the first time, we studied the anticancer potential of WA in TNBC cells at the miRNA level. Small RNA sequencing of WA-treated TNBC cells revealed differential expression of miRNAs compared to vehicle-treated cells, which indicate its miRNA expression modulation potential in test cells. Recently, a low expression of miR-181c-5p was reported in TNBC samples and cell lines, which indicates its tumor suppressive role in the breast cancer subtype. Moreover, the study found that increased miR-181c levels have potential to decrease cellular proliferation and induce apoptosis in TNBC cells [28]. The up-regulation of miR-15a inhibited cellular proliferation in TNBC cells. Shinden et al. (2015) showed a positive correlation between decreased disease-free/overall survival and low levels of miR-15a in TNBC patients [29]. Recently, it was shown that the higher expression of miR-15a decreases cellular proliferation and aggressiveness in TNBC cells [30]. Conversely, the up-regulation of miR-1275 and miR-326 was associated with the pathophysiology of TNBC [31,32]. In the present study, we found that WA treatment in TNBC cells significantly reversed the expression profile of miR-181c-5p, miR-15a-5p, miR-1275, and miR-326 compared to vehicle-treated cells. This is evidence that WA has potential to exert anticancer potential in TNBC cells by modulating the expression profile of tumor suppressors and oncogenic miRNAs. Furthermore, time- and concentration-dependent WA-mediated induced expression of miR-181c in TNBC cells and the corresponding lower cellular viability in test cells indicate that WA targets TNBC cells by up-regulating the expression of miR-181-5p (Figure 3 and Figure 4).

The GO analyses of the WA-treatment-induced DEMs target genes revealed alterations in the expression of genes of pathways important in their anticancer effect, such as cell differentiation, protein serine/threonine kinase activity, and apoptotic process, etc. (Figure 2). Previously, the effect of WA on the mRNA expression profile was reported, which showed the enrichment of differentially expressed gene enrichment in the mTOR and MAPK signaling pathways [25]. Furthermore, it has been reported that the NF-ĸB/mTOR and PI3/Akt signaling pathways mediated apoptosis induction in the WA-treated breast TNBC cells [27]. In the present study, we also found enrichment of WA-treatment-induced DEMs target genes in the mTOR, MAPK, and PI3/Akt signaling pathways. Moreover, the enrichment of a higher number of target genes in MAK and PI3/Akt signaling pathways indicates the possible modulation of these pathways and, thereby, the associated cancer hallmarks by WA-induced miRNAs. The data indicate that our data is consistent with the published reports.

Although the role of miR-181c-5p in mitochondrial gene modulation in normal physiological conditions and some disease conditions was reported, its potential role in breast cancer cells is still not documented [33,34]. Thus, the potential role of miR-181c in mitochondria membrane potential and ROS generation alteration in TNBC cells were studied. In the present study, we found that WA significantly up-regulated miR-181c levels in TNBC cells. As the primary source of energy for aggressively growing cancer cells, mitochondria are one of the most important targets in cancer therapies. The data suggest that the loss of mitochondrial membrane potential (MMP) is involved in apoptosis induction in cancer cells [35]. The intact MMP allows the cationic JC-1 to cross into the mitochondria. Red fluorescence is indicative of the presence of J-aggregates in mitochondria, which are present in healthy cells. The breakdown of the MMP causes the JC-1 dye to become monomeric and accumulate in the cytoplasm of apoptotic cells, giving them a green fluorescence. Previously, it was shown that WA has the ability to disrupt the MMP in breast cancer cells [36]. The present work is the first to report that WA disrupts MMP by increasing the level of miR-181c in TNBC cells (Figure 5B Panel I). In the present study, WA showed concentration-dependent ROS generation induction potential in TNBC cells, which further increased by using the WA and miR-181c mimic co-treatment strategy. WA IC_30_ and miRNA-181c mimic co-treatment increased ROS generation compared to the WA IC_50_ co-treatment group, which might be due to the death of test cells at the combination treatment concentration (Figure 5 Panel II). Hahm et al. (2011) reported that WA induces apoptosis by increasing the mitochondria-derived reactive oxygen species in breast cancer cells at a micro molar concentration [36]. Thus, even though WA-mediated ROS generation was reported previously in breast cancer cells, the present work is the first to report WA-induced ROS generation by increasing the level of miR-181c in TNBC cells. These findings have important translational implications because they suggest that the anticancer response to WA may be attenuated in the presence of antioxidants.

The up-regulation of miR-181c in ovarian cancer cells increases the drug sensitivity in drug-resistant cells by inducing apoptosis [37]. In a different study, the forced expression of miR-181c temozolomide sensitivity by Ribophorin II (RPN2) inhibition mediated apoptosis induction in glioblastoma cells [38]. Similarly, the apoptosis-induction-mediated anticancer potential of miR-181c was reported in endometrial, hepatocellular, and cervical squamous cell carcinoma [39,40,41]. The apoptosis-induction potential of miR-181c is well documented in different cancers, but little information is available for breast cancer. Recently, it was shown that miR-181c has potential to induce apoptosis in TNBC cells. A previous study showed the apoptosis-induction potential of WA in TNBC cells at the micro molar concentration [18,28,42]. Although the stemness/self-renewal inhibition efficacy of WA at the miRNA level in breast cancer stem-like cells is demonstrated by our research group, here, for the first time, we report the miRNA-mediated anticancer effect of WA in breast cancer cells. In the present study, we found that miR-181c-induced apoptosis was related to morphological changes in TNBC cells that were augmented with the increasing concentration of WA in a dose-dependent manner (Figure 5A Panel I). These results indicate that WA may exert apoptosis-related morphological changes in TNBC cells by augmenting the levels of miR-181c. Furthermore, the increased reduction of PARP and Bcl2 gene expression and the increased expression of caspase 3, caspase 8, and the BAX gene in the WA+miR-181c mimic treated group compared to alone treatment indicate that WA exerts apoptosis induction by elevating miR-181c levels in TNBC cells. Moreover, the increased caspase 3/7 activity in WA+miR-181c mimic treated group also indicates the miR-181c level augmentation mediated apoptosis potential of WA in TNBC cells.

**Figure 8:**
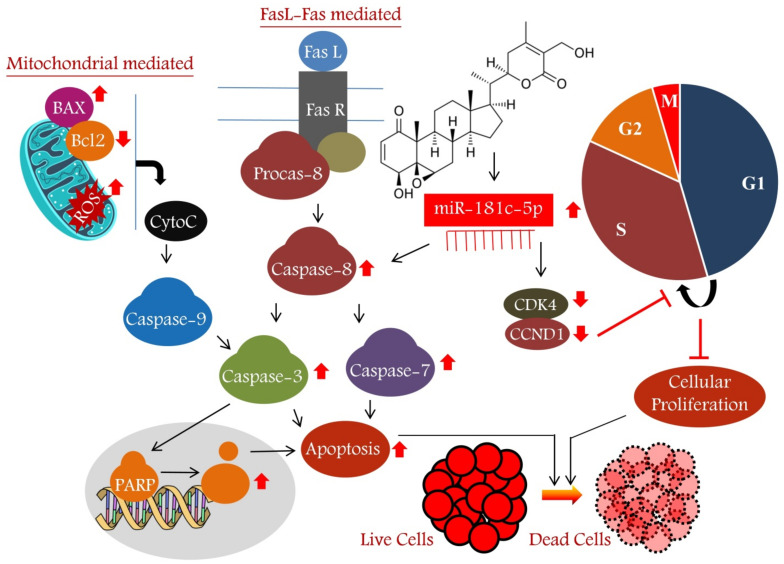
The microRNA-mediated anticancer mode of action of Withaferin A in triple-negative breast cancer cells. Withaferin A treatment increased miR-181c-5p expression, which reduces cellular proliferation and induces apoptosis in MDA-MB-231 cells. CytoC—Cytochrome C; Fas—Fas ligand; Fas R—Fas receptor; Procas—Procaspase; ROS—Reactive oxygen species; G2—G2 phase; M—M phase; G1—G1 phase; S—S phase.

The FasL-Fas or extrinsic pathway is one of the strategies to induce cell apoptosis in cancer cells. In the present study, the co-treatment of WA and miR-181c mimic significantly elevated cleaved caspase 8 and 3 levels and increased caspase 3/7 activity in TNBC cells (Figure 6). These results indicate that WA induces extrinsic apoptosis pathways in TNBC cells by elevating miR-181c levels. The previous reports on the phytochemical-mediated increase in caspase-3/8 and BAX and the decrease in the Bcl2-level-induced apoptosis in breast cancer cells corroborates our findings [43,44]. The mitochondrial- or intrinsic pathway-mediated apoptosis induction is associated with the alteration in MMP, cytochrome c release, and caspase 9 and 3 activation. Additionally, Bcl2 and BAX proteins also play a pivotal role in intrinsic apoptosis pathways. Although the present study was not primarily focused on identifying the type of apoptotic pathway, the results (increased cleaved caspase 3 and BAX expression, as well as caspase 3/7 activity) indicate that WA induces intrinsic apoptosis pathway by elevating miR-181c levels in TNBC cells. Previous reports on mitochondria targeting mediated apoptosis induction in breast cells by WA further strengthen the hypothesis [36]. Figure 8 depicts the miR-181c-5p mediated anticancer mode of action of Withaferin A in TNBC. Previously, it was reported that withanolide E has the potential to inhibit MDA-MB-231 and SK-Br-3 TNBC cells at 0.24 and 0.71 µM IC_50_ concentrations, respectively. Thus, in the future, it would be interesting to examine the efficacy of miR-181c-5p and withanolide E co-treatment in TNBC cells, which might produce a more pronounced anticancer effect [45]. Moreover, the studies on the combined effect of Withaferin A+miR-181c-5p and PARP inhibitor(s) or CDK4/6 inhibitor(s) are also warranted to find new therapeutic strategies for TNBC.

## 5. Conclusions

To the best of our knowledge, for the first time, the present study introduces the miRNA-mediated anticancer activity of Withaferin A in a triple-negative breast cancer in vitro model. Withaferin A treatment inversely altered the expression of oncogenic and tumor-suppressive miRNAs in TNBC cells compared to vehicle-treated cells. Withaferin induces cellular toxicity in TNBC cells by increasing the level of miR-181c-5p and inhibits cell-cycle G1/S transition by lowering CDK4 and Cyclin D1 protein levels. Furthermore, Withaferin A induces the mitochondria and Fas ligand–Fas-mediated intrinsic apoptotic pathway in triple-negative breast cancer cells by up-expressing miR-181c-5p levels. In conclusion, the present work indicates the increased efficacy of Withaferin A in combination with miR-181c-5p, which could act as a novel strategy to target triple-negative breast cancer.

## Data Availability

Small RNA next generation sequencing (NGS) raw and processed data were deposited in NCBI’s Gene Expression Omnibus (GEO), and the assigned GEO Series accession number of the submitted data is GSE183395. The other data that support the findings of this study are available in the supplementary material of this article.

## Supplementary Materials

The following are available online at https://www.mdpi.com/article/10.3390/metabo13010029/s1, Supplementary File S1–S5. Table S1: List of miRNA primers and their sequences; Table S2: List of mRNA primers and their sequences; Table S3: List of differentially expressed miRNAs among the test groups; Table S4: The Log2FC and p values of the top five up and down-expressed miRNAs. Table S5: List of miRNA target genes for significant differentially expressed miRNAs.
